# Role of Decidual Natural Killer Cells in Human Pregnancy and Related Pregnancy Complications

**DOI:** 10.3389/fimmu.2021.728291

**Published:** 2021-08-26

**Authors:** Xiuhong Zhang, Haiming Wei

**Affiliations:** ^1^Department of Genetics, School of Life Science, Anhui Medical University, Hefei, China; ^2^Hefei National Laboratory for Physical Sciences at Microscale, Division of Molecular Medicine, The Chinese Academy of Sciences (CAS) Key Laboratory of Innate Immunity and Chronic Disease, School of Life Sciences, University of Science and Technology of China, Hefei, China; ^3^Institute of Immunology, University of Science and Technology of China, Hefei, China

**Keywords:** human pregnancy, decidual natural killer cells, inflammation, anti-inflammation, maternal-fetal interface, immune tolerance, pregnancy complications

## Abstract

Pregnancy is a unique type of immunological process. Healthy pregnancy is associated with a series of inflammatory events: implantation (inflammation), gestation (anti-inflammation), and parturition (inflammation). As the most abundant leukocytes during pregnancy, natural killer (NK) cells are recruited and activated by ovarian hormones and have pivotal roles throughout pregnancy. During the first trimester, NK cells represent up to 50–70% of decidua lymphocytes. Differently from peripheral-blood NK cells, decidual natural killer (dNK) cells are poorly cytolytic, and they release cytokines/chemokines that induce trophoblast invasion, tissue remodeling, embryonic development, and placentation. NK cells can also shift to a cytotoxic identity and carry out immune defense if infected *in utero* by pathogens. At late gestation, premature activation of NK cells can lead to a breakdown of tolerance of the maternal–fetal interface and, subsequently, can result in preterm birth. This review is focused on the role of dNK cells in normal pregnancy and pathological pregnancy, including preeclampsia, recurrent spontaneous abortion, endometriosis, and recurrent implantation failure. dNK cells could be targets for the treatment of pregnancy complications.

## Introduction

Successful pregnancy in humans is reliant on a series of critical events: embryo implantation, decidualization, placentation, and parturition. Each of these events is crucial to a good pregnancy outcome.

At the onset of human pregnancy, the blastocyst hatching from the zona pellucida adheres and implants into the maternal uterine endometrium. There is a high prevalence of implantation failure after natural conception and in *in vitro* fertilization (IVF) therapy (1). Uterine stromal cells that surround the implanting embryo differentiate into large secretory decidual cells (“decidualization”). The decidua provides nutritional support and an immune-privileged matrix to the embryo before establishment of a functional placenta (2). After implantation, the trophectoderm of the implanted blastocyst proliferates and differentiates rapidly into two main subpopulations: syncytiotrophoblast (the multinucleated epithelium of the villi responsible for nutrient exchange and hormone production) and extravillous trophoblast (EVT; which invades the uterine endometrium of the mother through “placentation”). The placenta provides sufficient nutrients and is a barrier to immune tolerance for the developing fetus (3). If the fetus is at term, parturition is initiated by inflammatory and endocrine signals, which drives quiescent uterine tissues to an active labor state, and promotes contractions (4, 5). These physiological events in pregnancy are inflammatory processes, and a balance of pro- and anti-inflammatory factors is required for remodeling of intrauterine tissue, feto-placental growth, and parturition throughout gestation (6).

Natural killer (NK) cells play a crucial part in the initiation and resolution of inflammation (7), and they are detected in all phases of pregnancy (8–10). NK cells are cytotoxic innate lymphoid cells, and were first discovered thanks to their ability to kill tumor cells, and later found to also kill pathogen-infected cells (11). In humans, conventional NK cells are present in peripheral blood (pNK cells) and are distributed widely throughout the body. pNK cells are divided primarily into two subtypes: cluster of differentiation CD3^−^CD56^dim^CD16^+^ cells and CD3^−^CD56^bright^CD16^−^ cells. It has been found that 90–95% of pNK cells, CD56^dim^ NK cells, have potent cytotoxicity and high expression of CD16. CD56^bright^ NK cells are best known for producing diverse types of cytokines with weak cytolytic activity (12). In addition to pNK cells, in humans NK cells are also found in peripheral tissues, such as the liver, lungs, skin and uterus, and are termed “tissue-resident NK” (trNK) cells. Most trNK cells are the subset of CD56^bright^ NK cells. The latter exhibit different signatures that are related to their tissue of origin, and show high expression of CD69, CD103, and CD49a, which have been used to identify trNK cells (13). Decidual NK (dNK) cells are a specialized type of trNK cells found at endometrial decidual tissue, and display many unique phenotypic and functional characteristics compared with pNK cells and trNK cells (14).

Herein, we review the emerging knowledge about human dNK cells. We focus specifically on the phenotypes and functions of NK cells under human physiological and pathological pregnancy conditions.

## Characteristics And Subtypes Of dNK Cells In Human Pregnancy

dNK cells comprise ~70% of immune cells in the first-trimester decidua (8). Vento-Tormo and colleagues identified three main subsets of dNK cells (dNK1, dNK2 and dNK3), which all co-express the tissue-resident marker CD49a together with proliferating NK cells from isolated first-trimester decidual cells by single-cell RNA-sequencing (15). Compared with dNK2 and dNK3 cells, dNK1 cells show higher expression of killer cell immunoglobulin-like receptor (KIR) genes (human leukocyte antigen (HLA)-C receptor: KIR2DS1, KIR2DS4, KIR2DL1, KIR2DL2 and KIR2DL3) and Leukocyte Immunoglobulin-Like Receptor B1 (ILT2, an HLA-G receptor which is expressed only by the dNK1-cell subset). HLA-G and HLA-C are expressed primarily on EVTs of fetal origin. The interaction between HLA-C and HLA-G molecules with their receptors on dNK1 cells contributes to trophoblast invasiveness, vascular remodeling, and maintenance of a local microenvironment of immune tolerance (16, 17). In addition, dNK1 cells contain more cytoplasmic granule proteins (perforin 1, granulysin, granzyme A GZMA and GZMB) which provide immunity against placental infection and the enzymes involved in glycolysis. Studies have shown that adaptive NK cells from human cytomegalovirus (HCMV)-seropositive individuals exhibit enhanced glycolytic metabolic profiles relative to that in canonical NK cells (18). Increased expression of glycolytic enzymes in dNK1 cells suggests that they may be responsible for supporting repeated pregnancies. dNK2 and dNK1 cells co-express activating killer cell lectin-like receptor C2 (NKG2C) and NKG2E (activating receptors on NK cells) as well as NKG2A receptors (inhibitory receptor on NK cells) for HLA-E molecules, which indicates similar functions between dNK2 and dNK1 cells (19). dNK2 cells also expresses high levels of X-C motif chemokine ligand 1(XCL1) which is known as lymphotactin. Whereas XCR1, the receptor of XCL1, is expressed on EVTs and dendritic cells. Bottcher et al. proved that NK cells producing XCL1 chemokines promote cDC1 recruitment by surface receptor XCR1 on cDC1 (20). The recognition and combination of XCL1–XCR1 suggests that dNK2 cells mediate recruitment of EVTs and dendritic cells at the fetal–maternal interface. dNK3 cells are in low proportion and show high expression of chemokine ligand 5 (CCL5). C-C motif chemokine receptor 1 (CCR1, the receptor for CCL5) is expressed by EVTs. Sato et al. reported that chemokine-CCR1 interactions induced migration of the EVTs to maternal tissue (21), which suggests a role for dNK3 cells in regulating EVT invasion. Those findings suggest the importance of dNK cells in the first-trimester decidua, and in particular, dNK1 plays a dominant role in early pregnancy.

Whether the phenotypic and functional properties of dNK cells remain unchanged throughout pregnancy is an important concept. Zhang and colleagues measured expression of the activation receptors, degranulation capacity, cytokine expression, and proliferation of human dNK cells during the first and second trimesters. They found that the number and cytokine expression [e.g., interferon (IFN)-γ, vascular endothelial growth factor (VEGF) and interleukin (IL)-8] of dNK cells were not significantly different in the first trimester and second trimester. dNK cells in the second trimester showed higher expression of active receptors (NKp80 and NKG2D), but limited degranulation capacity of dNK cells in comparison with that in the first trimester. Zhang and colleagues speculated that inhibition of dNK-cell function may lead to two mechanisms during the first and second trimesters: suppression of activating-receptor levels in the first trimester by trophoblasts and disengagement of receptor–ligand coupling in the second trimester (9). Likewise, de Mendonca Vieira and colleagues investigated the function, and phenotype, of dNK cells in a term pregnancy. By comparison with pNK cells and first-trimester dNK cells, they suggested that the proportion of term-pregnancy dNK cells among CD45+ cells were significantly lower and dNK cells in a term pregnancy had an increased degranulation response, but lower capacity to respond to human CMV-infected cells. They also identified that expression of a set of NK cell receptors, and found that term pregnancy dNK had fewer HLA-C receptors (KIR2DL1, KIR2DL2/3, and KIR2DS1) but more HLA-E receptor NKG2D compared with first trimester dNK. NKG2A, and NKG2C were no significant changes between the two dNK types. Term pregnancy EVT had the highest expression levels of HLA-G. They also detected the expression of HLA-G receptors in term pregnancy dNK, KIR2DL4 and ILT4 were no significant differences between first trimester dNK and term pregnancy dNK. In addition, a series of genes including *IFN-γ*, *GZMH*, *interferon gamma receptor 1(IFNGR1), CD69, integrin subunit beta 2(ITGB2), NKp8*0, was upregulated by term-pregnancy dNK cells compared with that in first-trimester dNK and pNK cells (10). The different receptors and gene-expression profile of term-pregnancy dNK cells indicates a distinct type of NK cells, and the specific function of term-pregnancy dNK cells remains to be determined. But previous reports have stated that dNK cells progressively disappear from mid-gestation onwards and virtually are absent at term (22, 23). A possible explanation for the discrepancy may be due to the different detection methods (the early studies identified NK cells by staining cytoplasmic granules). Granulated leukocytes were rare after 20 weeks (24). Later studies identified NK cells distribution in third trimester by immunostaining CD56. While there are a proportion of agranular CD56+ uNK cells in third trimester, and these cells may be overlooked.

In repeated pregnancies, dNK cells display unique phenotypic properties. They show increased expression of NKG2C and ILT2 and enhanced production of IFN-γ and VEGFα, and display a special type of innate memory: these cells are termed “pregnancy-trained dNK cells” (25). Greater expression of VEGFα and IFN-γ can better support vascularization and initiate remodeling of endometrial vasculature in development of the placental bed, so pregnancy-trained dNK cells are more beneficial for subsequent pregnancies.

There is also evidence that dNK cells can be induced for a senescent phenotype by interacting with HLA-G from trophoblasts during pregnancy (26, 27). The NK cells of senescent phenotype would produce pro-inflammatory factors and pro-angiogenic factors that contribute to for trophoblast invasion and spiral artery remodeling (28).

In humans, NK cells are also divided into four subsets according to relative expression of the surface markers CD27 and CD11b. CD27 has been indicated as a marker for dividing mature NK cells into two functionally distinct subsets (29). The CD11b has been identified as a marker of human NK cells maturation (30). CD11b^+^CD27^−^ NK cells exhibit high cytolytic ability; CD11b^−^CD27^+^ and CD11b^+^CD27^+^ NK cells have the best ability to secrete cytokines; CD11b^−^CD27^−^ NK cells display differentiation potential (31). For dNK cells, ~60% are CD11b^−^CD27^−^ NK cells and >20% are CD27^+^ NK cells (32).

## The Role Of dNK Cells in Normal Pregnancy

### Regulation of Uterine Natural Killer (uNK) Cells by Ovarian Hormones

Progesterone and estrogen are the two main ovarian hormones involved in regulation of the menstrual cycle and establishment and maintenance of pregnancy (33). Some studies have revealed that uNK cells accumulate extensively around spiral arterioles in the mid-secretory-phase endometrium and early-pregnancy decidua in accordance with increasing levels of ovarian-derived estrogen and progesterone (34). Those findings indicate that the recruitment and/or expansion of uNK cells may be regulated by these hormones. It is known that NK cells express receptors for specific chemokines and can be induced to migrate to specific tissues in response to several chemokines (35). Some studies have suggested that estrogen and progesterone induce expression of chemokines C-X-C motif chemokine ligand 10 (CXCL10) and CXCL11 in the human endometrium, whereas pNK cells and uNK cells show high expression of specific receptors for these chemokines. Therefore, progesterone and estrogen have indispensable roles in regulating the recruitment of NK cells into the uterus (36). This phenomenon could also explain the increase in uNK-cell number during the menstrual cycle.

Progesterone is a major driver of decidualization. Some studies have shown that progesterone can stimulate endometrial stromal cells to secrete IL-15 to promote the proliferation and differentiation of uNK cells in an indirect manner due to the absence of progesterone receptor on uNK cells (37, 38). Besides, estrogen and progesterone can regulate the function of uNK cells. It has been reported that human uNK cells can express glucocorticoid receptor (GR) which is a member of the superfamily of nuclear receptors, and that progesterone cross-reacts significantly with GR. Guo et al. found that progesterone could inhibit the IFN-γ production of uNK cells *via* GR (39), and induce immune tolerance during early pregnancy. Estrogens regulate uNK-cell migration directly and promote secretion of CCL2 from uNK cells, which facilitates uNK cell-mediated angiogenesis (40).

### dNK Cells in Implantation and Decidualization

uNK cells are the major leukocytes in the endometrium. They comprise ≤30% of total lymphocytes in the endometrium at the mid-secretory phase (also called the “window of implantation”) and 70–80% of the total leukocyte population in the decidua from early pregnancy, which suggests that NK cells have a crucial role in implantation and decidualization (41). Paradoxically, implantation in humans and rodents is reliant on a proinflammatory mechanism. This inflammatory reaction is essential for implantation. Most of the accumulated evidence indicates that IVF patients with recurrent implantation failure (RIF) subjected to endometrial biopsy exhibit a substantial improvement in their chance to conceive (42). During the window of implantation, the uterus is “primed” under the action of ovarian hormones to release proinflammatory cytokines and chemokines, including IL-8, IL-15, IL-6, CXCL10 and CXCL11 (36, 43), which activate and recruit large populations of decidual immune cells to the endometrium at the time of implantation. Of these, 65–70% are uNK cells. Successful implantation is dependent upon an implantation-competent embryo achieving invasion into the receptive endometrium to establish a blood supply for the conceptus. uNK cells play a crucial role in this process with a recent study indicating that dNK cells act as biosensors of low-quality human embryos. Low-quality blastocysts that failed to implant secreted lower levels of hyaluronidase 2 (HYAL2), a member of hyaluronidases family that regulates hyaluronan (HA) size at tissue. Low levels of HYAL2 and high levels of high molecular weight HA (HMWHA) inhibit dNK cells-mediated clearance of senescent decidual cells. Hence, dNK cells determines endometrial fate at implantation (44). Further, in the first weeks of pregnancy (the period of embryo implantation in human), these trophoblast cells express soluble HLA-G (sHLA-G) which is bound by the NK cells receptor KIR2DL4, activating a proinflammatory/proangiogenic response which is beneficial to the establishment of receptive endometrium (45). Brighton et al. indicated that decidualization induced acute senescence in a subpopulation of ESCs. The senescence-associated secretory phenotype drives the initial auto-inflammatory decidual response linked to endometrial receptivity. As pregnancy progress, dNK cells eliminate senescent decidual cells to regulate endometrial rejuvenation and remodeling upon embryo implantation, and maintain the homeostasis of the endometrium (46). Expression of prokineticin 1 secreted by uNK cells is increased during the mid-secretory phase of the menstrual cycle, and increased further in early pregnancy. It has been proposed as a marker of a receptive endometrium because it regulates expression of a series of implantation-related factors, including leukemia inhibitory factor, IL-11, and prostaglandins (47, 48). Interestingly, their involvement in the development of a receptive endometrium in humans is crucial whereas, in mice, mature NK cells do not appear in the uterus before implantation (49). In addition, elevated uNK cells in women who have repeated early pregnancy losses contribute to pathological elongation of the window of endometrial receptivity which permits abnormal or delayed embryos to implant (50).

Endometrial decidualization in humans is triggered whether or not there is a conceptus. During pregnancy, once decidualization is initiated, the state of the endometrium is translated from a phenotype of acute inflammatory initiation to an anti-inflammatory phenotype. This process is accompanied by the massive infiltration of immune cells, including NK cells, which are termed dNK cells. The number of NK cells begins to increase around LH+3 (pre-decidualization) with large numbers densely scattered throughout the stroma in the late secretory (decidualization). NK cells coexist with the decidual tissue and are also observed in ectopic decidua (49). These findings provide valuable hints that the NK cell may be associated with the decidualization. Differentiation of endometrial stromal cells (ESCs) into specialized decidual cells is the most typical feature of decidualization. Several hormones, cytokines, growth factors, and morphogens are involved in regulating this process (2). Zhang et al. reported that dNK cells facilitated ESC decidualization by secreting IL-25 (51). Recent studies have reported that the dNK cell from early miscarriage decidual tissues induce AEA (endocannabinoid anandamide) production by ESCs (52). AEA plasma levels are higher in women suffering miscarriage (53) and notably AEA has been shown to impair decidualization *in vitro* (54). The dNK cell from miscarriage cases also secrete higher level of TNF-*α*, which inhibits ESCs decidualization by decreasing the decidual markers prolactin (PRL) and insulin-like growth factor binding protein-1(IGFBP-1) (52). A study observed that decidual stromal cells (DSCs) displayed increased autophagy during decidualization, and accelerated the residence and enrichment of dNK cells during normal pregnancy. Depletion or absence of NK cells resulted in adverse outcomes (reduced number of embryos implanted, increased embryo loss, and angiogenesis disorders) in pregnant mice, and emphasized the importance of NK cells in the establishment and maintenance of normal pregnancy (55, 56). Broadly speaking, the process of decidualization also include spiral artery remodeling, and the role of dNK cells on spiral artery remodeling will be discussed below.

### dNK Cells in Placentation and Fetal Development

Following implantation and decidualization, the second important stage of pregnancy is initiated: rapid growth of the placenta and the growth and development of the fetus. Trophoblast invasion and vascular remodeling are the most critical moments during placentation. Reduced invasion of trophoblasts and vascular conversion results in poor placental perfusion, which is thought to be the underlying primary defect of common disorders of pregnancy (e.g., recurrent miscarriage, preeclampsia and fetal growth restriction) (57). During the placental formation, the role of dNK in regulating extravillous trophoblast (EVT) invasion is dependent on gestational age (58). Several studies suggest that dNK cells at 8–10 weeks of gestation mainly produce angiogenic growth factors which are associated with spiral artery remodeling in early pregnancy (59). Later between 12–14 weeks, dNK cells mainly produce cytokines (IL-8 and INF-γ inducible protein, IP10) that stimulate EVT invasion by increasing MMP-9 secretion and reducing EVT apoptosis (58). It is known that excess EVT invasion can endanger placenta and mother. However, dNK can also secrete a range of cytokines, TNF-α, TGF-β and IFN-γ, inhibit EVT excessive invasion in later stages (60, 61).

When trophoblasts complete their invasion (~20^th^ week of pregnancy), the number of dNK cells begins to decrease (8). To support the demands of the growing fetus, uterine spiral arteries (SAs) must remold to a wide diameter and be capable of transporting adequate nutrition and oxygen to the fetus (62). Although trophoblasts are involved in SA remodeling, the initial stages, including loss of vascular smooth muscle cells (VSMCs) and breaks in the endothelial-cell layer, occur in the absence of EVTs but in the presence of lymphocytes (63). Accumulating evidence suggests the direct influence of dNK cells on SA remodeling. dNK cells infiltrating near human SAs express a wide range of MMPs which can initiate early breakdown of the extracellular matrix of SAs in the absence of EVTs (64). One study demonstrated that VSMC loss in SA remodeling occurs *via* migration away from the vessel wall, and not apoptosis (65). Dedifferentiation of VSMCs is an important feature of migration. Yang and colleagues showed that the uterine decidual *niche* (including dNK cells) modulates the progressive dedifferentiation of human VSMCs in SAs (66). The factors secreted by dNK cells, including chemokines, cytokines and vasoactive factors, such as IL-8, TGF-β, angiopoietin-1/2 (Ang1/2), and VEGF-C, initiate destabilization of vascular structures and, thus, SA transformation (59, 67, 68). As mentioned above, sHLA-G from EVT induces a senescent state in NK cells capable of participating in SA remodeling by secreting a series of factors (TNF-α, IL-1β, IFN-γ, IL-6, IL-8 (28).

A role for dNK cells in SA remodeling in human disease has also been noted. Reduced numbers of dNK cells have been demonstrated in patients with pre-eclampsia and intrauterine growth restriction (IUGR), which are associated with poor remodeling of SAs and reduced trophoblast invasion in the decidua (69). In addition to the role of NK cells in placentation, they can also promote the growth and development of the fetus. Fu et al. identified a CD49a^+^ subset of dNK cells that promoted fetal development by secreting growth-promoting factors, including pleiotrophin (PTN)and osteoglycin (OGN), before establishment of the placenta in humans and mice. Ultimately, a deficiency in these growth factors leads to growth restriction by abnormal development of bone in offspring (70). Zhou et al. demonstrated that the transcription factor PBX homeobox 1 can directly regulate transcriptional expression of growth-promoting factors in dNK cells and drive fetal growth (71).

### Regulation and Function of dNK Cells at the Maternal–Fetal Interface

At ~5 weeks after implantation, the human placenta is formed from trophoblasts of fetal origin and decidua of maternal origin. The placenta constitutes an interface connecting the mother and the fetus: the maternal–fetal interface (72). This is a unique process, the mother, placenta, and the fetus with paternal antigen are symbiotic processes. The maternal immune system must accept the semi-allogeneic fetus while preserving immune defense against pathogens, and the predominant immunological feature of this phase is induction of an anti-inflammatory state.

Many efforts have been made to explain the mechanism of maternal–fetal interface immune tolerance. The maternal–fetal interface is composed mainly of fetal trophoblasts, maternal DSCs, and decidual immune cells (23). In the first trimester, human decidual leukocytes are primarily NK cells (∼70%) and macrophages (∼20%) (24). In addition to an intrinsic lower cytotoxicity of decidual CD56^bright^CD16^−^ NK cells, several studies have indicated that dNK cells interact with HLA ligands (e.g., HLA-G, HLA-C and HLA-E) expressed on EVTs to depress the cytotoxic capability of dNK cells. HLA-G is a non-classical HLA class-I molecule, and is uniquely expressed in EVTs (73). At the maternal–fetal interface, dNK cells show high expression of the inhibitory receptors KIRs, such as KIR2DL1, KIR2DL2/L3 and ILT2, which recognize HLA-G to inhibit NK-cell cytotoxicity (73). Beyond that, HLA-G-induced immune tolerance has been found to occur by a peculiar cell biological process: “trogocytosis” which is defined for lymphocytes can extract surface molecules through the ‘immunological synapse’ from interacting cells (74). One study demonstrated that primary human dNK cells can uptake HLA-G proteins produced by primary human EVTs (75), and internalized HLA-G correlates with the very low cytotoxicity of freshly isolated dNK cells (76). One possible explanation may lie in the fact that EVT–NK-cell synapses are inhibited during HLA-G endocytosis and endo-lysosomal signaling events, and inhibition of these synapses weakens the cytotoxicity of NK cells (76). Another non-classical HLA class-I molecule expressed by trophoblasts, HLA-E, can regulate the cytotoxicity of dNK cells by interacting directly with the inhibitory receptors CD94/NKG2A (77). HLA-C (a classical HLA class-I molecule) also weakens the cytotoxicity of NK cells by interacting with the inhibitory receptors KIRs (78).

T-helper (Th)17 cells are a critical lineage of proinflammatory Th cells involved in development of autoimmune disease. Excess Th17 cells directly cause fetal loss *in vivo* (79). Wei and colleagues indicated that decidual CD56^bright^CD27^+^ NK cells “dampened” inflammatory Th17 cells by secreting IFN-γ to promote immune tolerance and successful pregnancy (79). Indoleamine 2,3-dioxygenase (IDO) is a key metabolic enzyme responsible for tryptophan degradation (80). IDO is produced widely at the fetal–maternal interface (81). Ban et al. indicated that trophoblast-derived IDO could downregulate expression of NKp46 and NKG2D and reduce the cytotoxicity of pNK cells; it may also contribute to maintaining dNK-cell cytotoxicity at a low level, and play an important part in maintenance of normal pregnancy (82). T-cell immunoglobulin domain and mucin domain-containing molecule-3 (Tim-3) is a newly defined regulatory factor. Tim-3 can modulate the balance of Th1 cells/Th2 cells (83). Li et al. were the first to detect Tim-3 expression in dNK cells, Tim-3^+^ dNK cells displayed decreased cytotoxicity due to producing less perforin than Tim-3^-^ dNK cells (84). In addition, Huang et al. found that microRNA-30e expression was upregulated in the decidual tissues in healthy pregnant women, and was involved in immune tolerance at the maternal–fetal interface by increasing expression of KIR2DL1 and decreasing expression of NKp44 to suppress dNK-cell cytotoxicity (85). At the maternal–fetal interface, CXCL16 secreted by trophoblasts induces the polarization of macrophages towards the M2 phenotype. M2 macrophages attenuate NK-cell cytotoxicity by decreasing IL-15 secretion, which has important roles in the differentiation, maturation, and survival of NK cells to establish immune tolerance (86).

Curiously, although dNK cells display low cytotoxicity, they show high expression of cytotoxic granules, such as perforins, granzymes, granulysin and several NK-activating receptors, including NKp46, NKp44, NKp30, and NKG2D, compared with peripheral-blood CD56^bright^ NK cells (87). *In utero* infection by viruses (Zika, HCMV), bacteria (*Listeria monocytogenes*) and parasites (*Toxoplasma gondii* and *Plasmodium* species) causes fetal defect/loss, premature labor, and IUGR (88–91). However, the rate of vertical transmission is quite low in the first trimester, which coincides with high numbers of dNK cells within the placental bed (14). These observations indicate that dNK cells can play an important part in maintaining maternal–fetal tolerance under physiological conditions, but also present cytotoxicity under infection. Some studies have observed that individuals who carry more activating KIR also have a significantly improved outcome after viral infections (e.g., HCMV, human immunodeficiency virus, human papillomavirus) (92, 93). Siewiera et al. provided the first evidence that dNK cells can clear HCMV-infected DSCs (94) and that HCMV-infected EVTs cannot be cleared (75). A recent study suggested that dNK cells killed bacteria in trophoblasts by transferring granulysin without killing placental cells (95). Conversely, viruses can induce expression of activating ligands (e.g., major histocompatibility class I polypeptide–related sequence A (MICA) and MICB) on the surface of infected cells that bind directly to activating NK receptors and promote NK-cell cytotoxicity (96). NK-derived IFN-γ is crucial in antimicrobial immunity because it activates macrophages and promotes differentiation of Th1 cells (97). Furthermore, understanding the mechanisms that regulate the switching of dNK cells between immune tolerance and immunity at the maternal–fetal interface may contribute to the development of novel strategies to limit pathogen-induced placental infections.

### NK Cells in Parturition

Parturition is an inflammatory process. During late pregnancy, extensive evidence suggests that reproductive tissues (myometrium, placenta, cervix, and fetal membranes) can secrete chemotactic factors (e.g., CXCL8, CXCL10, CCL2 and CCL3), and are responsible for the selective recruitment of circulating maternal leukocytes (innate and adaptive) to these tissue (98–100). These leukocytes, along with reproductive-tissue cells, secrete proinflammatory mediators, including cytokines (IL-1, IL-6, IL-8, and TNF), MMPs, and prostaglandins, which induce cervical effacement/dilatation and rupture of the membranes, leading to labor and delivery of the baby [77]. It is thought that premature activation of this proinflammatory pathway can lead to a breakdown of tolerance of the maternal–fetal interface and, subsequently, can result in preterm birth (101). The latter is a major determinant of neonatal mortality and morbidity (102).

Several studies in humans and mice have reported the important role of neutrophils, macrophages, T cells, and B cells during parturition. With the progression of pregnancy, dNK cells lose granules in the cytoplasm, which indicates that a functional shift is needed at late gestation for parturition (103). Some have studies indicated that NK cells are also involved in regulating labor. Pique-Regi et al. used single-cell RNA-sequencing to profile the placental villous tree, basal plate, and chorioamniotic membranes of women with labor at term and those with preterm labor. They observed that the chorioamniotic membranes, basal plate, and placental villi largely contained lymphoid and myeloid cells, including T cells, NK cells, and macrophages. In addition, they reported that expression of the single-cell signatures of NK-cells and activated T-cells was upregulated in women with spontaneous labor at term compared to gestational-age matched controls without labor (104). NK T cells are a unique lymphocyte subset that express the markers and characteristics of the adaptive and innate immune system. St Louis et al. identified activated NK T-like cells to be more abundant in the decidual basalis of women who underwent preterm labor, and demonstrated that *in vivo* NK T-cell activation led to preterm labor by inducing a maternal systemic proinflammatory response (105). However, the regulatory mechanisms of NK cells in labor remain are not clear.

## The Role Of dNK Cells In Pathological Pregnancy

### Preeclampsia (PE)

PE is a serious complication of pregnancy that manifests as maternal hypertension and proteinuria. This pregnancy complication affects 5–8% of all pregnancies worldwide, and is a major cause of maternal and perinatal morbidity and mortality worldwide (106, 107). PE is subdivided into early-onset (starts before 34 weeks) and late-onset PE (starts after 34 weeks). Early-onset PE has a close relationship with inadequate placentation and placental ischemia. However, the pathological placenta is a result of incomplete invasion by trophoblasts and SA remodeling (108, 109). As mentioned above, dNK cells have critical roles in regulating SA remodeling with trophoblasts by producing a series of cytokines and chemokines. Hence, dNK-cell dysfunction has been implicated in PE initiation. Some studies have indicated that there is higher risk of women suffering PE if they carry alleles for KIR AA genotype (lacking most or all activating KIR) on maternal NK cells when the trophoblast expresses HLA-C2 (a much stronger inhibitory effect when binding to KIR2DL1 inhibitory receptors) (110, 111). Thus, excessive inhibition of uNK cells after trophoblast binding, as well as reduced production of angiogenic factors and cytokines, is detrimental to placentation and arterial transformation (112).

HLA-G can protect trophoblasts from dNK-cell lysis, Pazmany et al. showed that patients with severe PE have reduced expression of HLA-G (113). The CD94/NKG2A receptor on dNK cells binds to HLA-E molecules to provide an overall inhibitory signal of preventing cell lysis. A recent study suggested that *NKG2A* ablation in mice caused abnormal vascular remodeling in pregnancy. They also found that a 7% greater relative risk associated with the maternal HLA-B allele (most of which encode activating receptors, KIR2DS1, 2, 3, 5, and KIR3DS1) that fail to educate NKG2A^+^ NK by analyzing whole genome sequence of 7,219 PE case (114). Natural cytotoxicity receptors (e.g., NKp44, NKp46 and NKp30) are unique markers to NK cells that regulate cytokine production and cytotoxicity. A significantly reduced percentage of NKp46^+^ NK cells in the peripheral blood of women with PE compared with that of women not suffering from PE can be observed 3–4 months before PE onset (115). Abnormal placental TGF-β response is related to pathological development of PE (116). Zhang et al. reported that higher levels of TGF-β produced by decidual Treg cells suppressed dNK cell function by downregulating IFN-γ/IL-8/CD107a expression, and also selectively modulated the proportions and function of specific dNK subsets in preeclamptic decidua, which may have a direct effect on the pathogenesis of PE (117).

Additionally, there are a number of studies showing that altered numbers of uNK cells are associated with PE. Some studies have reported that the numbers of dNK cells are significantly higher in PE compared with normal pregnancies (118, 119) although other studies have reached the opposite conclusion (69, 120, 121). The contradictory results may be due to the differences in the specimen origin (e.g. decidua basalis *versus* placental bed biopsies), sample size, test or analytic methods used. More meaningful results may be obtained by detecting the changes in the different NK cells subtype and NK cells functions rather than measuring absolute changes in cells number. In a PE model in BPH/5 mice, Sones et al. showed lower levels of uNK cells in the decidua (122). Based on those studies, abnormal activation of NK cells in PE could be a target for improving treatment of PE.

### Recurrent Pregnancy Loss (RPL)

According to American Society of Reproductive Medicine criteria, RPL is the experience of at least two or three spontaneous miscarriages before the 24th gestational week. About 50% of RPL cases are caused mainly by chromosome abnormalities, endocrine disorders, uterine defects, and infections (123, 124). The cause of the other 50% of cases is not known, and such cases are referred to as “unexplained RPL” (uRPL). These unexplained cases are associated with immunologic dissonance (125). A series of studies have shown that abnormal numbers and subsets of NK cells may be associated with uRPL. Most of the studies have suggested that higher concentrations of uNK cells are detected in women with uRPL than that in healthy fertile women (126–129). In other studies, despite a lack of differences in the proportion of uNK cells between controls and women with RPL, women with RPL showed a significant decrease in the subset of CD56^bright^CD16^−^ NK cells, and significantly increased populations of cytotoxic CD16^+^ uNK cells expressing high levels of the cytotoxicity receptors NKp46, NKp44, and NKp30 (129, 130). Ebina et al. revealed pre-pregnancy increased activities of pNK cells to be associated with pregnancy loss (131). Intravenous immunoglobulin (IVIG) has been shown to be efficacious treatment of uRPL, particularly in patients with increased numbers of NK cells. IVIG can reduce the cytotoxicity of pNK cells *in vitro* and *in vivo* (132, 133). CD49a expression on dNK cells regulates the early adhesion and migration of dNK cells into trophoblasts, and limits their cytotoxicity by downregulating expression of perforin, granzyme B, and IFN-γ. dNK cells from women who underwent RPL had lower levels of CD49a and higher expression of perforin, granzyme B, and IFN-γ compared with dNK cells from age-matched healthy controls (134). Those studies suggest that the cytotoxicity of uNK cells is higher in RPL than that in healthy controls. Guo et al. provided evidence of lower KIR2DL4 expression on dNK cells and lower HLA-G expression on trophoblasts in patients with RPL, which led to impairment of the pro-invasion and pro-angiogenesis functions of dNK cells (135). This may be a possible mechanism explaining RPL. Another subset of uNK cells in the endometrium and decidua, IL-22-producing NK cells, has been found to be higher in number in uRPL than that in infertile women (136). Therefore, accumulating evidence suggests that uNK cells are profoundly dysregulated in uRPL, and that evaluating the activity of NK cells may be a predictive marker for RPL.

### Endometriosis

Endometriosis is a common gynecological disease which affects 10% of all women of reproductive age. It causes chronic pelvic pain, dysmenorrhea and infertility (137). Endometriosis is characterized by endometrial tissue outside the uterine cavity. Ectopic endometrium is found most commonly in the pelvis and is thought to arrive by retrograde menstruation (138). The pathophysiology of endometriosis is incompletely understood, but accumulating evidence indicates that this disease could be an immune-related chronic inflammatory process (139).

The current consensus is that the function of immune-related (including NK) cells is impaired. The role of NK cells in the removal of menstrual debris and endometrial fragments that are likely to reach the peritoneal cavity by retrograde menses has been studied extensively (140). Most studies have focused on the change of number of pNK cells and NK cells in peritoneal fluid. There is no difference in the number of pNK cells and peritoneal fluid in women with endometriosis compared with those without this disease (141). However, the cytotoxicity of pNK cells and NK cells in peritoneal fluid from endometriosis patients is reduced significantly by display of increased expression of inhibitory receptors (e.g., KIR2DL1) and diminished expression of activating receptors (e.g., KIR2DS1, and CD94/NKG2A) compared with that in healthy women (142–144). Those results suggest that low cytotoxicity of pNK cells and NK cells in peritoneal fluid may reduce clearance of ectopic endometrial fragments in the peritoneal cavity. However, there have been very few studies on uNK cells in endometriosis. One study discovered that the percentage of uNK cells increased progressively from the proliferative phase. The highest number was in the late secretory phase in the eutopic endometrium of women with endometriosis, with no difference in fertile healthy women. However, the percentage of uNK cells in ectopic lesions remained significantly low throughout the menstrual cycle (34), enabling the survival of endometrial cells in ectopic lesions.

Infertility is a common complication of endometriosis. It is estimated that 50% of women with endometriosis are infertile (145). Hence, the change of microenvironment in the eutopic endometrium in endometriosis may be associated with infertility. One study reported that the number of CD16^+^ and NKp46^+^ (cytotoxic uNK cell-surface receptors) uNK cells was increased significantly in the endometrium of women with endometriosis who were infertile or experienced recurrent pregnancy loss, compared with that in fertile women (126). Those data suggest that increased activity of uNK cells may not be conducive to establishment of normal pregnancy. Besides, an increased number of immature uNK cells has been found in women with endometriosis-associated infertility compared with those without endometriosis (146). This phenomenon may also explain the infertility associated with endometriosis. Thus, the differences between the number of uNK cells in patients with endometriosis *versus* those without endometriosis needs further exploration to judge which changes increase the risk of infertility in these patients.

### RIF

RIF is defined as the failure to achieve a clinical pregnancy after transfer of at least four high-quality embryos in a minimum of three fresh or frozen cycles in a woman under 40 years of age (147). Approximately 10% of women after IVF embryo transfers experience RIF. Multiple factors may contribute to RIF, including disturbance of the endometrial microenvironment, which significantly influences embryo implantation during the establishment of pregnancy. As discussed above, uNK cells are the major leukocyte population within the endometrium at the time of implantation, which suggests that uNK cells should be focused upon if exploring RIF pathogenesis. Using flow cytometry and immunohistochemistry, some studies have reported that that the number of uNK cells increases during the peri-implantation period in the endometrium of women with RIF (148–150). However, a recent a study showed that the number and distribution of uNK cells relative to endometrial arterioles was not significantly different in women with RIF compared with that in women in whom embryo implantation was successful following IVF (151). A meta-analysis showed no differences in IVF outcomes among women with or without increased number of uNK cells (128). These paradoxical results may be due to differences in laboratory protocols or sampling time in the endometrium. Chen et al. observed that isolated CD56^+^ uNK cells from women with RIF produced a lower level of angiogenic factors (e.g., VEGF and PLGF) compared with that in normal controls with proven fertility (41). Similar to preeclampsia, the risk of RIF is related to the haplotypic polymorphism of KIR genes. Alecsandru et al. found that women with the maternal KIR AA haplotype were more susceptible to suffering RIF after IVF treatment than those with the KIR AB haplotype or KIR BB haplotype (152). In addition, NKp44 expression on uNK cells was upregulated significantly in RIF patients, and suggested that the high cytotoxicity of NK cells may be one of the causes of RIF (115). Perhaps due to the difficulty in obtaining suitable endometrial samples, there have been few studies on the role of uNK cells in RIF.

## Conclusions

**Figure 1:**
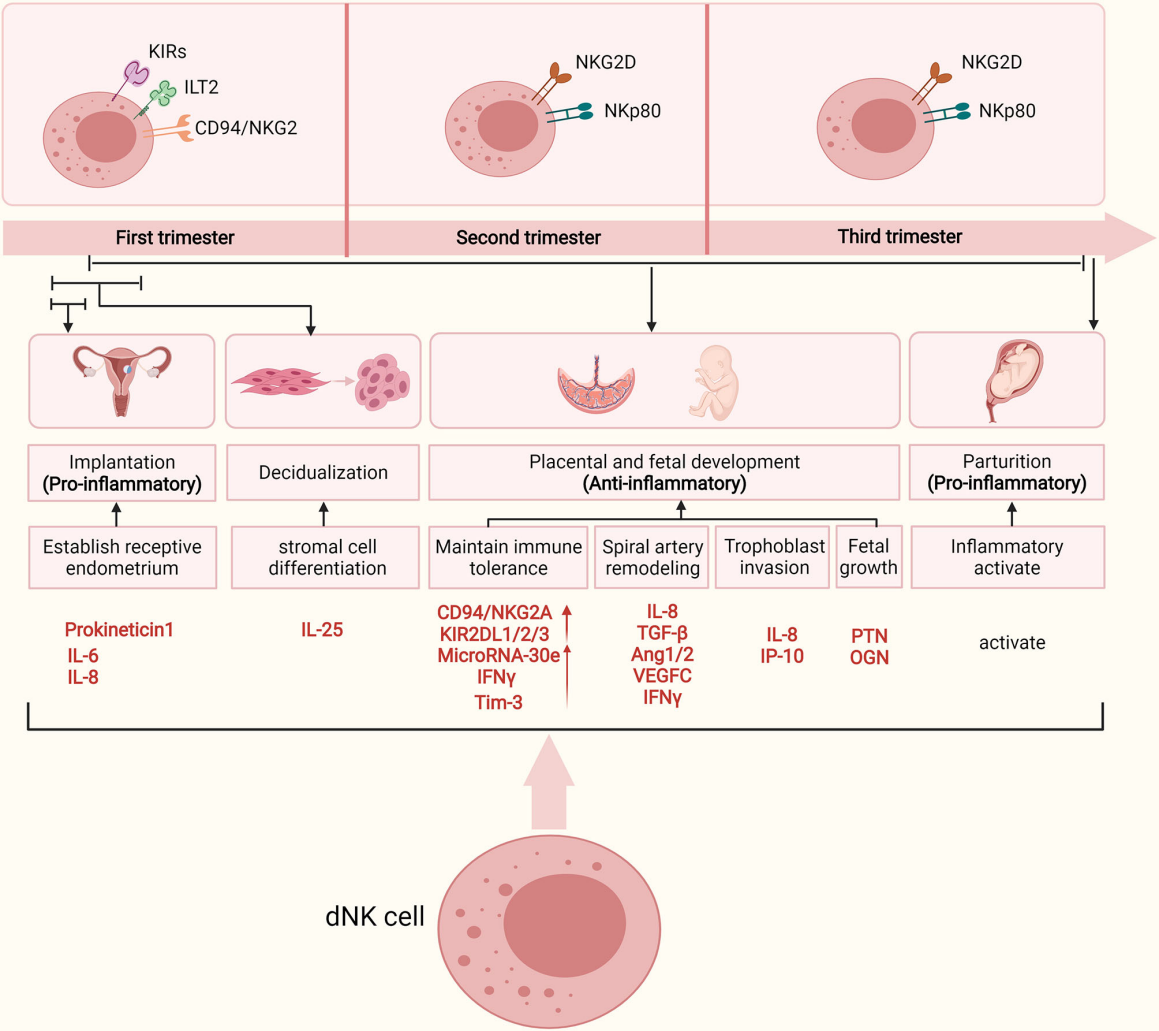
The primary roles dNK cells during key stages of human pregnancy and major subtypes of dNK cells in different trimesters. During early pregnancy, uNK cells highly express prokinetincin1, a marker of receptive endometrium, and proinflammatory factors (IL-6, IL-8) facilitating the embryo to implant into endometrium. dNK cells facilitate ESC decidualization by secreting IL-25. During human placental and fetal development, inhibitor receptors expressed on dNK cells (such as KIRs, CD94/NKG2) interact with HLA ligands expressed on EVTs to depress the cytotoxic capability of dNK cells, thus maintaining immune tolerance in maternal-fetal interface. Up-regulation of microRNA-30e, Tim-3 and IFN-γ in dNK cells also contributes to the immune tolerance in maternal-fetal interface. dNK cells participate in the remodeling of spiral arteries by secreting chemokines, cytokines and vasoactive factors, such as IL-8, TGF-β, IFN-γ, Ang-1/2, VEGF-C. dNK cells can also promote fetal development through secreting PTN and OGN. At late gestation, the activation of NK cells leaded to labor by inducing a maternal systemic pro-inflammatory response. In addition, the major dNK cell subtype classified by receptor expression are shown for the different trimesters. The figure was created with biorender.com.

**Figure 2:**
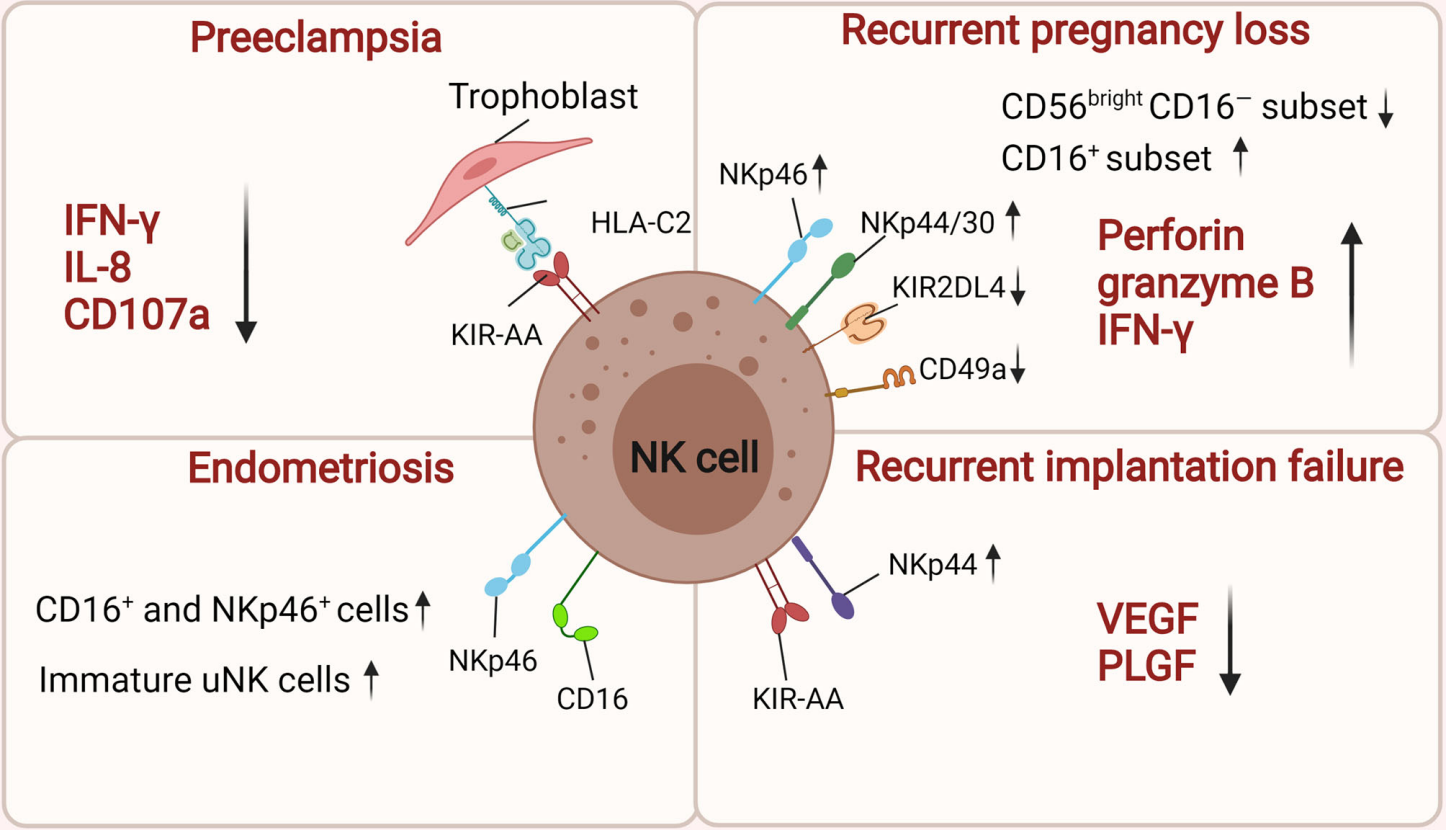
The roles of uNK cells in related pregnancy complications. There is high risk of women suffering PE if they carry alleles for KIR AA genotype on maternal NK cells when the trophoblast expresses HLA-C2. The inhibition of dNK activation by downregulating IFN-γ, IL-8 and CD107a contributes to the onset of preeclampsia. uNK cells from women with RPL have low levels of CD49a, KIR2DL4, and high expression of NKp46/44/30, perforin, granzyme B, and IFN-γ. Moreover, their cytotoxic CD16^+^ uNK cells populations is significantly increased while the CD56^bright^CD16^−^ NK cells subset is significant decrease. In women with endometriosis, the number of CD16^+^, NKp46^+^ uNK cells and immature uNK cells was significantly increased in the endometrium. Women suffering RIF express low levels of angiogenic factors (VEGF and PLGF) and those with a maternal KIR AA haplotype are more susceptible to suffering RIF after IVF treatment. The figure was created with biorender.com.

This review emphasizes the important role of dNK cells throughout pregnancy (**Figure 1**). These cells undertake different functions during several critical stages of pregnancy. In the early stages of pregnancy, dNK cells do not present a cytotoxic response against the semi-allogeneic embryo. dNK cells interact with HLA ligands expressed on EVTs to depress the cytotoxic capability of dNK cells and mediate immune tolerance at the maternal–fetal interface. In addition, they are key regulators in the early stages of pregnancy because they secrete several cytokines, thereby having a fundamental role in vascular remodeling, trophoblast invasion, and embryonic development. NK cells can also shift to cytotoxic behavior and undertake immune defense upon *in utero* infection by pathogens. At late gestation, dNK cells are reactivated to break immune tolerance and induce parturition. However, the underlying molecular basis of dNK cells for the transition from a weak cytotoxic status to robust status in different stages has yet to be revealed. Further investigation is required on how these dNK subtypes may change during pregnancy and which factors determine their mechanism of transition. Furthermore, the abnormal number and activity of NK cells can lead to various reproductive diseases, such as RSA, PE, Endometriosis, RIF (**Figure 2**). Therefore, understanding the normal physiology of pregnancy will help to reveal the pathogenesis of pregnancy complications. This will be meaningful for the treatment and management of the disease.

## Author Contributions 

All authors contributed to the article and approved the submitted version. XZ drafted the manuscript and figures. HW edited/reviewed the article.

## Funding

This work was supported by the key project of the National Key Research and Development Program of China (#2018YFC1003900) and the National Natural Science Foundation of China (#81930037, U19A2024).

## Conflict of Interest

The authors declare that the research was conducted in the absence of any commercial or financial relationships that could be construed as a potential conflict of interest.

## Publisher’s Note

All claims expressed in this article are solely those of the authors and do not necessarily represent those of their affiliated organizations, or those of the publisher, the editors and the reviewers. Any product that may be evaluated in this article, or claim that may be made by its manufacturer, is not guaranteed or endorsed by the publisher.
